# The Private Partners of Public Health: Public-Private Alliances for Public Good

**Published:** 2009-03-15

**Authors:** Sharon McDonnell, Carol Bryant, Jeff Harris, Peggy A. Hannon, Marci Kramish Campbell, Ano Lobb, Jeffrey L. Cross, Barbara Gray

**Affiliations:** Prevention Research Centers Program, Division of Adult and Community Health, National Center for Chronic Disease Prevention and Health Promotion, Centers for Disease Control and Prevention; Florida Prevention Research Center at USF, Tampa, Florida; University of Washington, Seattle, Washington; University of Washington, Seattle, Washington; University of North Carolina, Chapel Hill, North Carolina; Dartmouth Medical School, Barre, Vermont; American Cancer Society, Atlanta, Georgia; Centers for Disease Control and Prevention, Atlanta, Georgia

## Abstract

**Objective:**

We sought to convey lessons learned by the Centers for Disease Control and Prevention's (CDC's) Prevention Research Centers (PRCs) about the value and challenges of private-sector alliances resulting in innovative health promotion strategies. Several PRCs based in a variety of workplace and community settings contributed.

**Methods:**

We conducted interviews with principal investigators, a literature review, and a review of case studies of private-sector alliances in a microbusiness model, a macrobusiness model, and as multiparty partnerships supporting public health research, implementation, and human resource services.

**Results:**

Private-sector alliances provide many advantages, particularly access to specialized skills generally beyond the expertise of public health entities. These skills include manufacturing, distribution, marketing, business planning, and development. Alliances also allow ready access to employee populations. Public health entities can offer private-sector partners funding opportunities through special grants, data gathering and analysis skills, and enhanced project credibility and trust. Challenges to successful partnerships include time and resource availability and negotiating the cultural divide between public health and the private sector. Critical to success are knowledge of organizational culture, values, mission, currency, and methods of operation; an understanding of and ability to articulate the benefits of the alliance for each partner; and the ability and time to respond to unexpected changes and opportunities.

**Conclusion:**

Private-public health alliances are challenging, and developing them takes time and resources, but aspects of these alliances can capitalize on partners' strengths, counteract weaknesses, and build collaborations that produce better outcomes than otherwise possible. Private partners may be necessary for program initiation or success. CDC guidelines and support materials may help nurture these alliances.

## Introduction

Grant makers and grantees point out that there are three principal motivators for companies to take up a social agenda: values, strategy, and the pressure of regulation or litigation, either actual or threatened. If you get all three of those running at the same time, then you've got a chance to get something that lasts from one business cycle to the next.

— From *Working With the Business Sector: Pursuing Public Good With Private Partners* (1)

The practice of public health involves translating community needs into system responses that involve multidisciplinary and cross-sector alliances with political, medical, educational, economic, environmental, and social services. Flexibility and local specificity combined with high-quality, best-practice information from national and international sources can create innovative and effective public health programs. The Prevention Research Centers (PRC) program (www.cdc.gov/prc/about-prc-program/index.htm) was created to enable quality research for such programs. Managed by the Centers for Disease Control and Prevention (CDC), the PRC program has 33 centers across the United States. Each represents a collaboration of academic, public health, and community partners, working to explore new topics and approaches, conducting community-based participatory research (CBPR) and dissemination, and testing interventions to enhance public health. The PRCs' CBPR and efforts to sustain programs in diverse communities have led to alliances with nontraditional entities outside the health system, including the private sector.

Several PRCs have found that private-public alliances can contribute to a project's effectiveness by bringing specialized skills to work with niche problems and providing access to specific populations. The alliances can be time- and resource-intensive to nurture, but they capitalize on partners' strengths, compensate for weaknesses, and build a collaboration, the output of which is greater than the sum of its inputs (2,3).

An alliance can take many forms, including 2 organizations contributing equally to create a product for sale, or 1 organization providing services to another for no fee. Partnerships can include a spectrum of organizational types (eg, from private for-profit, nonprofit, government, and pseudogovernmental organizations) and service types (eg, mechanical production, technical support, advocacy, data analysis). Each member in an alliance brings its own culture, values, modes of operation, responsibilities, and constituents, along with its unique and specialized skills. Understanding these attributes is important because identity can be enigmatic. For example, the operations of a nonprofit organization, such as the American Cancer Society (ACS), may more closely resemble those of a private-sector, for-profit business than of a governmental or public health entity. The combination of attributes and the overall goals define an alliance and the activities it will perform.

We present 3 case studies that show different types of alliances between PRCs and the private sector: the first describes a multiparty alliance that guides employers in implementing and evaluating evidence-based chronic disease prevention services for employees in the workplace; the second illustrates a private-sector alliance by using a microenterprise model to address unemployment as an underlying determinant of health; and the third details an alliance to create and test innovative technology to improve worker safety. We also describe insights gained by 2 other PRCs in their work with private partners.

## Case Studies

### Increasing chronic disease prevention via the workplace: a multiparty partnership

Since 2002, the Health Promotion Research Center at the University of Washington (UW PRC) (http://depts.washinton.edu/hprc/) has partnered with the ACS to offer guidance about chronic disease prevention practices in workplaces (www.acsworkplacesolutions.com/). This strategy, based on an ecological model of health promotion, focuses interventions on the organization rather than the individual. In this case, employer practices are targeted as a means of improving employees' health behaviors (4). The partners developed, tested, and delivered ACS Workplace Solutions, a multifaceted program based on the *Guide to Community Preventive Services,* a CDC publication that evaluates evidence and provides recommendations about public health interventions (www.thecommunityguide.org). The program helps employers improve 5 categories of health promotion practices: health insurance benefits, health policy, workplace programs, health-promoting communication, and changes in employee health behaviors (5,6).

In a pilot study at 8 large employers in the Pacific Northwest, the UW PRC found that the program increased targeted preventive behaviors among employees from 38% at baseline to 61% at follow-up 13 months later (*P* = .02) (7). Based on these findings, the UW PRC and ACS streamlined the program to increase participation from small and medium-sized employers (8). The resulting program includes a Web-based questionnaire that employers can self-administer or request ACS help. ACS staff then generate tailored reports for the employers that give recommendations to improve practices. This briefer version of the program connects employers with ACS staff and services but offers limited face-to-face assistance or implementation support. The intervention, ACS Workplace Solutions Assessment, is provided free of charge to employers who offer access to their facilities and use of their employees' time for monitoring and follow-up.

By functioning as an alliance, all parties could maximize their resources and implement nationwide  assessment. In 3 years, ACS trained 853 staff nationwide to deliver and support the program. By 2008, when 471 employers had implemented the program, more than 2 million employees had been reached. As a large, private voluntary organization with activities in all 50 states, the ACS "brand" and its credibility with employers and their employees helped the PRC quickly gain access to an employee population large enough to allow robust evaluations. The PRC offered ACS scientific credibility and the research experience necessary to test the program's effectiveness. The program enabled small and medium-sized employers to increase their employees' use of prevention services in their health plans without incurring additional costs.

Outcomes of the nationwide implementation are still being assessed. At baseline, only 41% of the recommended employer practices were in place; influenza vaccination and cancer screening were the most common practices, at 56% and 52%, respectively. Next steps for the alliance include follow-up with a sample of employers that completed the brief intervention to determine whether they changed their practices and to compare the effectiveness of the full and streamlined versions of ACS Workplace Solutions.

### Microenterprise model: creating a private-sector retail business to benefit community health

HOPE (Health, Opportunities, Partnerships, and Empowerment) Works is a CBPR project that addresses social and economic empowerment and hope among low-income, racially and ethnically diverse women in rural North Carolina. This project of the University of North Carolina at Chapel Hill's Center for Health Promotion and Disease Prevention (UNC PRC) began as a result of community-based research findings that showed the need to address one of the fundamental causes of poor health in communities: underemployment and unemployment (www.hpdp.unc.edu) (9).

**Figure 1.**
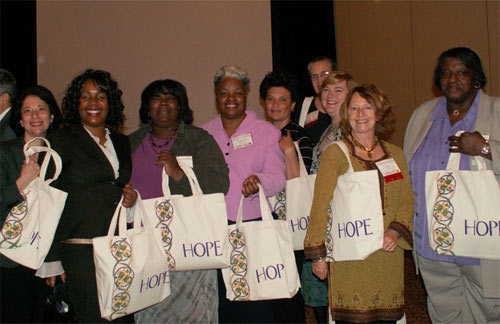
Threads of HOPE participants at the annual Prevention Research Centers program meeting held in Atlanta, Georgia, in March 2008 are shown holding canvas HOPE bags. The business was contracted to produce 300 bags for conference participants, and it has since been contracted to make 500 bags for a cancer survivor conference and 1,200 bags for the Centers for Disease Control and Prevention chronic disease conference in 2009.

After conducting formative research, the UNC PRC and the local community worked with a local nonprofit business association to train a team to conduct market research and develop plans for a new private-sector business. Collaboration with existing businesses provided training and mentoring in the basic skills required to plan and run a small business. This microenterprise intervention draws on the Grameen Bank model used in developing countries, in which women who live in poverty join social networking circles that provide resources, financial oversight, and education (www.grameenfoundation.org/who_we_are/) (10). The first venture emerging from this activity was Threads of HOPE, which produces high-quality tote bags for professional conferences (Figure 1). Products in development include ecologically friendly outdoor cushions. Beyond producing merchandise, Threads of HOPE serves the PRC's and the community's goals of providing training, mentoring, and networking to build business and employment opportunities and enhance local economic development.

Creating a private-sector retail business as a strategy to improve health represented an expanded role for academic researchers and public health practitioners who had little experience with business and economic development. However, the team leveraged support from the university, the local community, and CDC to obtain essential resources (such as seed funds for strategic planning) and to create robust social and technical support networks. Several nonprofit groups, including Good Work (www.goodwork.org) and the North Carolina Rural Center (www.ncruralcenter.org), also provided expertise in the planning process. Faculty and students from the North Carolina State University School of Design and local entrepreneurs were invited to participate in the project. Currently, the UNC-community partnership group has developed a business plan and applied for a foundation grant to fund infrastructure development, sewing machines, space, and fabric.

Future success is far from guaranteed. Approximately a quarter of new businesses fail within 2 years, and half fail within 4 (11). Among small businesses, survival is lowest for retail businesses, those with low capital (<$50,000), and those whose owners have less than a college education and little previous business experience. However, businesses started for personal reasons, that have multiple and older owners or partners, and that start up slowly as home-based enterprises, appear to have better longevity (11).

### Macrobusiness model: developing innovative technology to improve worker safety in Florida

In 1998 the University of South Florida Prevention Research Center (FPRC), in collaboration with a local community board and the Farmworker Association of Florida, identified occupational eye injuries as a priority health issue among Florida citrus workers (http://health.usf.edu/nocms/publichealth/prc/). Eye trauma and infections from contact with branches, combined with irritation and allergies from dust and chemicals, cause suffering, disability, and lost wages. Citrus companies were frustrated by employees' high medical costs and lost work time and were unable to increase the use of safety glasses to reduce injuries. Marketing research determined that although safety glasses could prevent 90% of eye injuries, less than 21% of workers used them.

**Figure 2.**
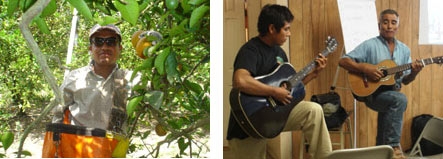
Left: Cesar Santes Valencia, a citrus worker and health promoter based in Immokalee, a settlement in the southwest tip of Florida, leans head-first from his ladder and picks rapidly. Protecting his eyes is a pair of safety glasses, held tight with a head strap. Right: The workers themselves come up with creative ways to reach their peers. Father-and-son orange pickers Cesar Perez Tiburcio, left, and Cesar Perez Muños, wrote a folk song about eye injuries and the importance of wearing safety glasses, which they played at a meeting of the Community Advisory Council.

A coalition of citrus pickers, citrus industry representatives, migrant farm worker advocates, and social service personnel launched a multifaceted community health promotion campaign. By hiring and training fewer than 3 dozen peer health promoters, safety glass usage increased from less than 1% to more than 30% among workers exposed to promoter programs, while those in crews without promoters did not change significantly (Figure 2) (12).

Despite this success, FPRC field research demonstrated that increased use of the safety glasses would not continue unless the glasses were improved. The principal improvement needed was a lens that would repel water and prevent fogging without distorting vision. With the assistance of a National Institutes of Health/CDC Small Business Innovative Research program grant, the FPRC developed a partnership with Reactive Innovations, a private technology firm, to manufacture and develop a coating that could be applied to lenses (www.reactive-innovations.com/). The prototype coating is undergoing field testing. If testing shows increased worker acceptability and safety glasses use while allowing the citrus workers to maintain quality work, the partners plan to develop a social marketing strategy to disseminate the safety glasses to citrus workers across Florida and possibly to other industries that need similar equipment.

The partners in this project included workers and the private, nonprofit, academic, volunteer, and government sectors. This diversity brought both strength and complexity that required each partner to learn about its other partners' culture and practices. The FPRC offered Reactive Innovations cultural information about a unique, hard-to-reach community that might not trust private-sector researchers. Reactive Innovations offered FPRC the ability to manufacture a product it otherwise would not have been able to create. As the developer of the lens coating, Reactive Innovations will hold the patent for it, which is an advantage for the private partner, but it could be a challenge for some university or public health partners unfamiliar with the risks and benefits of contractual agreements about product development and marketing (13).

## Themes

For government public health and university research partners unfamiliar with business, financial risk, and legal contracts, alliances with the private sector may be difficult to start. However, failing to create alliances that could benefit community health is unacceptable. In the following sections we summarize major themes and provide suggestions from the PRCs' experiences that may help public-private alliances to move forward.

### Culture, values, and mission

Creating public-private alliances involves all the challenges inherent in cross-cultural work. Differences between public and private organizations may concern identity, values, ethics, and operating principles. For example, academic institutions and public health entities may focus on process and be comfortable with projects progressing slowly. In contrast, businesses may focus on outcome and place high value on timeliness. The transparency and sharing of methods and outcomes ideal in academic research may be contrary to the culture of industry, in which trade secrets must be respected and intellectual property protected. Differences may be found in review and oversight, for example, legal review in a private organization may require changes to or even termination of a project that would survive scientific review intact. Conversely, the slow pace of meeting scientific peer review standards may be intolerable for a business partner not familiar with, or prepared for, such a process.

### Awareness of organizational "currency"

An organization's currency is closely tied to its mission and represents a unit of output that shows the degree to which the mission is being accomplished. An organization may value its currency above all else. Understanding the nature of an organization's currency extends beyond maintaining sensitivity toward its revenue-generating needs. For example, academic institutions value research, and research output is often measured as the number of research publications. Academicians rely on their peers in the scientific community to evaluate their work by the quality of its design, its thoroughness, and its intellectual and ethical rigor. The financial well-being of academic researchers depends on research grants, which may be awarded on the quality of past research. For businesses, currency may take the form of revenue (or profits or low cost in labor and health care), reputation, and a productive, stable workforce. In the nonprofit sector, currency may take the form of fundraising opportunities, projects that support mission, reputation, media coverage, credibility, filling a unique niche (for example, in health or technology), access to a hard-to-reach community, or a system or approach that might garner respect and status. A difference in currency can facilitate an alliance because the partners are not competing for the same resources. However, an alliance can be strained if one partner's currency is not valued as highly as another's or if aspects of currencies conflict. Successful alliances must be mutually beneficial, and it may not be possible to recognize and promote the benefits of collaboration to a prospective partner without knowing the currency it values. Ensuring that a relationship is mutually beneficial requires that partners understand the resources, technical skills, and the tangible or intangible assets each partner has to offer.

### Valuing unique attributes

Public health and academic entities bring unique attributes to an alliance. The PRCs' history of CBPR has resulted in strong, trusting relationships and social networks within their respective communities, relationships that business entities may value but not enjoy. For example, the FPRC was able to conduct market research with a migrant workforce that knew and trusted the PRC; private-sector researchers might not have been able to penetrate this population to nearly the same degree. PRCs also have public health expertise and research credibility, and they are perceived by communities as lacking the conflicts of interest that may be associated with private-sector businesses. To guard against tarnishing community ties when working with private partners, PRCs must be explicit about standards and responsibilities, and promote transparency to the community. Working with private- or business-sector partners local to a community may be advisable because the business may be particularly invested in its community standing. Private partners also may allow access to certain populations. For example, the workplace is an underused venue to access distinct populations and to deliver preventive health interventions. Similar to the UW and FPRC, the University of California Los Angeles/RAND PRC partnered with employers to reach specific populations (www.rand.org/health/centers/adolescent/). Its project used worksites as a venue for implementing health promotion and prevention programs. The Healthy Parents, Talking Teens program provided 8 hours of training in adolescent communications to 569 parents at 13 worksites and resulted in significant measured improvements in parent-child communications (14). To avoid missed opportunities for providing preventive services, public health practitioners must seek alternative venues for education, promotion, and even the provision of preventive services (15).

## Private-Sector Expertise

Public health agencies stand to benefit from the private sector's expertise in many areas, including market analysis and research, target audience assessment, marketing and product placement, distribution channels, and ongoing support (13). In addition, improved use and application of technology for core functions and innovation, already a cornerstone of private industry, may deserve more attention by public health entities. Although PRCs most often seek alliances with private enterprise for a project's final dissemination, partnership at early stages may be optimal. Effective dissemination requires an understanding of the intervention and its delivery, and a well-conceived and implemented dissemination strategy. The varied skills needed may require specialists from public health and from marketing. Some PRCs turn to private partners midway through a project or even at its outset. For example, formative research convinced the FPRC that success ultimately depended on a product that was beyond its expertise to develop, manufacture, or distribute. Locating a private partner with appropriate scientific and technical expertise allowed the project to continue. The PRC at Columbia University, working on an information technology project to develop patient-centered health information, needed the expertise of a private partner early in the project for software development and programming (www.healthyharlem.org/). The University of North Carolina Center for Health Promotion and Disease Prevention found the private sector helpful in developing human resources, mentoring, providing advice on local context, and setting up systems for a small retail businesses.

### Creating mutual benefit and reducing risk

Successful alliances must be mutually beneficial, and attracting partners may not be possible without understanding the resources, technical skills, and assets each one has to offer. Partners must plan time to build relationships at multiple levels of the allied organizations to solve problems and support daily operations. Solid knowledge of partners, nurtured through relationship-building, may help allay fears about the financial risks, legal issues (such as trademarks), product ownership, and other aspects of business. A jointly developed business plan should be part of the project timeline, and it should explicitly describe contractual relationships, responsibilities, and products, including issues of product ownership and funding over time (16). For example, the FPRC will not benefit if Reactive Innovations prices safety glasses out of reach of the citrus industry. Documentation should specify whether subcontracts and consultants will be involved so that the public health agency is prepared for the added complexity of these partners and relationships. The business sector may be familiar with such agreements, and public health entities must recognize that they represent community health interests that also need protection.

### Conclusion

Public-private alliances may be essential to advance some public health goals and to create sustainable community interventions. Such alliances require a willingness to think creatively about the benefit of nontraditional partnerships. Part of the challenge of creating health alliances with the private sector lies in the necessity of being able to expect, embrace, and respond to change. Each of the PRC projects required researchers to identify and adapt to alternative approaches. The CBPR process in itself guided researchers toward different targets and methods such as economic development.

CDC, in collaboration with other federal health agencies, is developing guidelines on how the public health sector can effectively work with the private sector and align private business interests with the public good (17). The PRCs' alliances with private-sector companies were fostered by CDC staff who recognized opportunities for collaboration, helped researchers develop mechanisms for the relationships, and provided support with the negotiation and maintenance of the alliances. This guidance about the public-private partnerships may also be important for state, local, and academic partners as they learn to how to form and maintain private sector alliances. The lessons learned by the PRCs may ultimately support the development of a multisector research agenda as well as professional and continuing education curricula for public health agencies and workers on strategies to involve the private sector in addressing societal and economic determinants of the population's health.
